# Overcoming staffing challenges when implementing a birth defects surveillance system: a Ugandan experience

**DOI:** 10.29392/001c.12503

**Published:** 2020

**Authors:** Joyce Namale-Matovu, Linda Barlow-Mosha, Daniel Mumpe-Mwanja, Dennis Kalibbala, Robert Serunjogi, jolly Nankunda, Diana Valencia, Evelyn Nabunya, Josaphat Byamugishat, Doreen Birabwa-Male, Margaret A Okwero, Monica Nolan, Dhelia Williamson, Philippa Musoke

**Affiliations:** * Makerere University – Johns Hopkins University Research Collaboration, Kampala, Uganda; † Makerere University – Johns Hopkins University Research Collaboration, Kampala, Uganda; ‡ Makerere University – Johns Hopkins University Research Collaboration, Kampala, Uganda; ** Makerere University – Johns Hopkins University Research Collaboration, Kampala, Uganda; †† Makerere University – Johns Hopkins University Research Collaboration, Kampala, Uganda; ‡‡ Department of Pediatrics and Child Health, Makerere University College of Health Sciences, Kampala, Uganda; *** Division of Global HIV and TB, US Centers for Disease Control and Prevention (CDC), Atlanta, GA, USA; ††† Department of Obstetrics and Gynaecology, Makerere University College of Health Sciences, Kampala, Uganda; ‡‡‡ Department of Obstetrics and Gynaecology, Makerere University College of Health Sciences, Kampala, Uganda; **** Mulago National Referral Hospital, Kampala, Uganda; †††† Division of Global HIV and TB, US Centers for Disease Control and Prevention (CDC), Uganda; ‡‡‡‡ Makerere University – Johns Hopkins University Research Collaboration, Kampala, Uganda; ***** Division of Global HIV and TB, US Centers for Disease Control and Prevention (CDC), Atlanta, GA, USA; ††††† Makerere University – Johns Hopkins University Research Collaboration, Kampala, Uganda; Department of Pediatrics and Child Health, Makerere University College of Health Sciences, Kampala, Uganda

**Keywords:** uganda, surveillance system, birth defects, staffing

Every year, 3–6% of infants worldwide are born with a serious birth defect.^1^ Approximately 3.3 million children under 5 years die from birth defects, and among those who survive, 3.2 million suffer with disabilities for life.^1^ Over 94% of all birth defects and 95% of deaths due to the birth defects occur in developing countries.^1^ Comprehensive, reliable data on birth defects are not available for most developing countries^2,3^ because implementing and maintaining birth defects surveillance systems requires substantial resources including staffing.

To provide accurate estimates of the prevalence of major external birth defects in Uganda, a hospital-based birth defects surveillance system is being implemented in four hospitals in Kampala, including one government hospital (Mulago) and three private, not-for-profit hospitals (Mengo, Nsambya and Lubaga).^4^ These hospitals were chosen because they represent approximately 55% of births (50,000 births/year) occurring in hospitals within Kampala.^4^ The surveillance collects demographic and basic medical information routinely collected through labor and delivery registry records, admission charts and/or antenatal cards or clinic records and is summarized in the surveillance record. In addition, all babies are examined by trained surveillance midwives for external birth defects. All mothers who deliver a baby with a major external birth defect(s), are asked if photographs can be taken of their baby to help with the diagnosis of the birth defect. Written informed consent is obtained before photographs are taken. Data from the surveillance study are collected electronically on a tablet.

This paper describes the different staffing approaches that were taken to set up the surveillance system, challenges encountered with each approach and actions taken to address these challenges. We also provide recommendations for other countries considering implementing a birth defects surveillance system in low-resource settings.

## OBTAINING STAKEHOLDER BUY-IN

Before the surveillance system could be implemented, buy-in from different stakeholders was needed. Initial consultative meetings were held with Uganda Ministry of Health officials, hospital directors/administration and Department of Obstetrics and Gynaecology heads to sensitize them about the proposed birth defects surveillance activities and obtain their support. Once support was obtained, sensitization and planning meetings were held with the Obstetrics and Gynaecology department staff including ward nurse/midwife in-charges, midwives and medical records staff that would be involved in the conduct of the surveillance.

## INITIAL CONCERNS OF PARTICIPATING HOSPITALS

During these consultative meetings, several concerns were raised. At the government hospital, the administration requested that additional midwives be hired to conduct surveillance activities so that the government midwives were not overburdened with surveillance activities and high volume of births (approximately 30,000 births per year; 1 midwife: 1040 births per year). The in-charge nurses/midwives voiced concerns about the addition of surveillance activities in the overcrowded delivery units including limited space to accommodate study staff and equipment; uncertainty about whether the responsibility of attending to delivering mothers in labor wards would be shared between government hospital midwives and surveillance midwives; and if they would be responsible for the supervision of both government hospital midwives and surveillance midwives.

The feedback from the nurses/midwives was obtained from approximately ten ward in-charges and their assistants through formal consultative meetings organized by the study leads who included but were not limited to: the Principal investigator, Co-investigator, Program manager, Hospital liaison coordinator (primary author), and the Data manager. As overall nurse/midwife leaders for five different maternity ward/ units, they were able to express their concerns about the addition of surveillance activities to the birth defects study team leads. In addition, meetings were held with over sixty maternity nurses/midwives who were not in-charges but were also working on the different maternity ward units under the overall in-charges. Focus group discussions were not conducted during the consultative meetings. The private, not-for-profit hospital (6,000-7,000 birth per year; 1 midwife: 520 births per year) administration decided that the existing hospital midwives would conduct the surveillance activities as this would benefit them by providing capacity building in research. However, they were also concerned about the increased workload of their staff, compensation and supervision reporting structure.

## SOLUTIONS

At the government hospital where the delivery workload was high, 24 additional surveillance midwives were hired to conduct surveillance activities. This number was based on the number of deliveries per day in each unit and number of midwives to cover all births 24 hours a day. They were paid by the surveillance study and worked alongside government hospital midwives to assist with routine deliveries. Government hospital midwives were not responsible for conducting any surveillance activities. All midwives (government and surveillance) were supervised by the existing ward nurse/midwife in-charges. Space for surveillance staff and equipment was obtained after negotiations with the hospital administration. At the private, not-for-profit hospitals, supervision of midwives for their regular duties did not change, and they were compensated for the additional work.

## TRAINING AND INTEGRATION OF SURVEILLANCE MIDWIVES

An intensive one-month training on surveillance procedures was conducted for all surveillance midwives. The training on surveillance procedures was conducted by experts from US Centers for Disease Control and Prevention (CDC) and the Uganda study leads who included the Principal investigator, Co-investigator, Program manager, Hospital liaison coordinator, and the Data manager together with the Regulatory team. The Uganda study leads had previously attended birth defect surveillance workshops conducted by International Clearinghouse for Birth Defect Surveillance and Research targeting health professionals in African countries in preparation for the surveillance and to train others.^5^

Training topics for one month included the study protocol, standardized operating procedures, infant examination, informed consent process, identification and characteristics of birth defects, data collection forms, android tablet use, Good Clinical Practice and Human Subjects Protection. The intensive one-month training consisted of fullday training sessions on working days from 8:30 am to 5:30 pm. A training schedule was in place and various facilitators gave sessions on different topics according to their area of specialty. After training, the new surveillance midwives were integrated into delivery wards at the government hospital in a staggered approach over a 4-month period. Afterwards, a pilot of surveillance activities was conducted for 3 months to identify and address any issues before actual implementation.

The pilot surveillance activities consisted of all surveillance activities so that issues of concern could be identified and addressed before project implementation. In addition to the roles mentioned in the background of this paper, during the pilot period, surveillance midwives also familiarized themselves with the hospital environment and tested all study tools, logs and tablet use. This period was used to edit standard operating procedures and study forms as well as practicing informed consent processes. The study leads assessed and re-assessed the working relationship between government and surveillance midwives as these interacted. The aim of the pilot was to see how best to integrate the surveillance system in the already existing labour ward services. Other objectives were to test the study tools (i.e. use of electronic tablet and study logs to collect data), ensure we had 24-hour midwife coverage and that all potential patients were included in the pilot.

**Figure 1.**
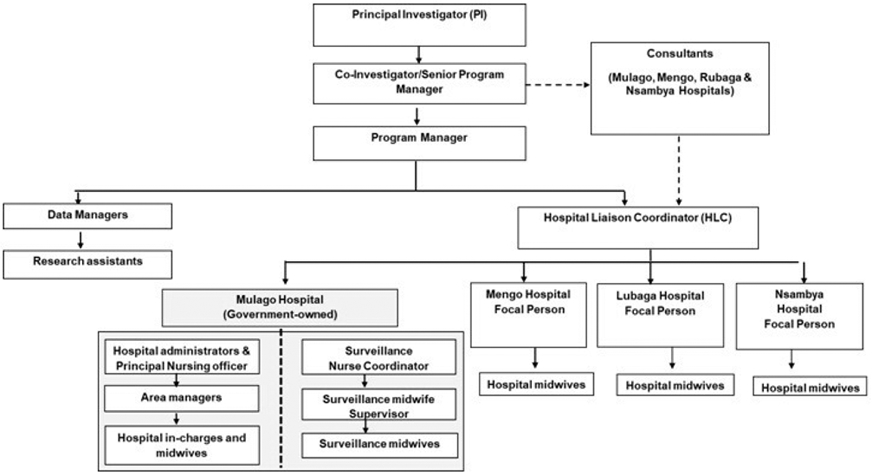
Birth defects surveillance system organogram.

At the private, not-for-profit hospitals, supportive supervision was conducted for one month by trained surveillance midwives from the government hospital and supervisory surveillance staff (Figure 1) to conduct on the job mentorship which continued whenever necessary.

## CHALLENGES ENCOUNTERED DURING INCORPORATION OF SURVEILLANCE ACTIVITIES

Several issues were identified during the integration in the delivery units at the government hospital: government midwives wanted monetary support for deliveries; the reporting structure/supervision hierarchy for government midwives was not sufficient; surveillance midwives needed supervision to ensure study activities were being conducted as per study protocol; the space allocated for surveillance staff and equipment was insufficient; and there was tension between the government and surveillance midwives.

In the private, not-for-profit hospitals, the midwives were concerned about conducting additional surveillance activities and not being well remunerated. These hospitals also had a high staff turnover rate or moving to other hospital units. Some midwives were also uncomfortable using the tablets for data collection.

## RESOLUTION

To address supervision hierarchy issues, a streamlined structure was created. In the government hospital, surveillance midwives would report to a midwife supervisor and nurse coordinator who were surveillance study staff while the government hierarchy remained unchanged (Figure 1). At the private, not-for-profit, a hospital focal person was identified to oversee surveillance activities. All surveillance-related issues identified at any of the four hospitals would be reported to the study hospital liaison coordinator who worked with hospital administration/staff to resolve.

Other activities were conducted at the government hospital to address challenges encountered including: negotiations with hospital administration to increase space; trainings to improve interpersonal relationships; defining specific roles of the surveillance midwives; sensitizing hospital staff about the surveillance; and modifying patient flow in specific high-volume delivery wards. These activities resulted in the government midwives working harmoniously surveillance midwives, with a reduction in the number of missed births (from 1.2% in October 2015 to 0.3% in January 2017) and improved supervisory structure. At the private, not-for-profit, the method of remuneration for surveillance activities was changed to performance-based to encourage midwives participate more in the study. An annual lump sum was given to the hospital to cover administrative costs and compensate midwives for conducting activities. The surveillance data manager provided technical support for tablet use, and over time the midwives became more comfortable with their use.

Although most issues were resolved, a few remained such as high midwife turnover rate at the private, not-for-profit hospitals, and delays in payment for the midwives. These payments required several approvals from different agencies within the private, not-for-profit hospitals, as well as verification of babies examined as a quality control measure, before midwives were paid. This process has been modified several times, but each reiteration still resulted in the midwives not being paid in a timely manner. To manage expectations, new midwives are informed that payment will be delayed for 2–3 months.

## LESSONS LEARNED

Implementation of a successful birth defects surveillance system into an existing hospital structure is a daunting task. The delivery volume at the participating hospitals may dictate the feasible staffing approach. Regardless of the approach taken, intensive preparatory activities and follow up are needed and should be planned for. These findings support the following interventions to ensure a smooth transition:

- Obtain buy-in from different stakeholders before start to sensitize them and obtain their support
- Hire study staff who understand the different hospital structures
- Include time for integration in the delivery wards for new surveillance midwives before activities begin to understand the hospital system and get to know hospital staff
- The number of additional midwives needed will be determined by the hospital workload including the number of units, births and available shifts
- Training of the surveillance midwives is of utmost importance and could take more than one month
- Pilot surveillance activities to address issues to ensure that procedures are correctly conducted before starting or expansion to other sites, regardless of whether the facilities are private or public.

Despite all challenges encountered, surveillance activities would not have been possible without the strong support among hospital administration/staff. The surveillance midwives were interested in learning about birth defects, new technology and participating in the study which built their capacity. Surveillance activities also have the potential to strengthen skills needed for routine work, such as conducting infant surface examinations and improving documentation of the exam by trained midwives and providing clinical staff knowledge about birth defect classification.
